# Mechanical Complications in Implant-Supported Prostheses: A Retrospective Analysis of Laboratory Intervention and Associated Factors

**DOI:** 10.4317/jced.63719

**Published:** 2026-01-28

**Authors:** Diego Gómez-Costa, Rocío Cascos-Sánchez, José Luis Antonaya-Martín, Noelia Rivas-Martín, Pablo Lastra-Prados

**Affiliations:** 1 Master’s Degree in Implant-Supported Prostheses, Rey Juan Carlos University (URJC); 2 Lecturer in the Master’s Programme in Implant-Supported Prostheses, URJC; 3 Doctor of Dentistry, Director of the Master’s Programme in Implant-Supported Prostheses, URJC

## Abstract

**Background:**

Mechanical complications in implant-supported prostheses frequently require dental laboratory intervention, resulting in prolonged treatment time and disruption of clinical workflow. Although prosthetic complications have been widely reported, limited evidence is available regarding those complications that specifically necessitate laboratory repair and their associated working time. Aim: To analyse mechanical complications requiring laboratory intervention in implant-supported prostheses and to evaluate their association with prosthesis type, complication category, and laboratory working time in a university-based clinical setting.

**Material and Methods:**

A retrospective observational study was conducted using clinical records of patients treated with implant-supported prostheses between 2018 and 2022. Cases presenting mechanical complications that required laboratory repair were included. Prosthesis type, complication category, complication severity, and laboratory working time were recorded. Descriptive statistics and multivariable analyses were performed to explore associations between variables.

**Results:**

Material fracture was the most frequent complication, particularly in bar-retained implant-supported overdentures. Single implant-supported crowns were more frequently associated with lack of adaptation. High-severity complications required significantly longer laboratory working time than medium-severity complications (mean 17.9 vs. 6.9 days; p &lt; 0.001). Prosthesis type and complication severity were independently associated with laboratory repair duration.

**Conclusions:**

Mechanical complications requiring laboratory intervention differ according to prosthesis design and have a significant impact on laboratory working time. These findings provide relevant information for treatment planning, maintenance strategies, and patient communication, although incidence of complications cannot be inferred due to the absence of denominators.

## Introduction

Implant-supported prostheses are widely used for the rehabilitation of partially and fully edentulous patients. High implant survival rates have been consistently reported; however, long-term treatment success is influenced by the mechanical performance of the prosthetic reconstruction (1 , 2). Mechanical complications include material fracture, loss of retention, screw loosening, decementation, framework failure, and problems related to prosthesis adaptation. While some of these events can be managed chairside, others require removal of the prosthesis and dental laboratory intervention, resulting in increased treatment time and impact on patient care (3 , 4). Systematic reviews have identified veneering material fracture as one of the most frequent mechanical complications, particularly in complete-arch restorations and overdentures (1 , 5). However, most studies report complication rates without distinguishing between minor events and those requiring laboratory repair. Data regarding laboratory working time associated with prosthetic complications remain limited (6). University-based clinical environments provide structured documentation and follow-up but may involve variability related to operator experience and laboratory workflows (7). Analysis of complications in such settings may provide insight into real-world maintenance demands. The aim of this study was to analyse mechanical complications requiring laboratory intervention in implant-supported prostheses and to evaluate their association with prosthesis type and laboratory working time.

## Material and Methods

1. Study Design and Ethical Considerations This retrospective observational study was conducted at the Rey Juan Carlos University Dental Clinic. The study protocol was reviewed and approved by the Institutional Ethics Committee. All data were anonymised prior to analysis in accordance with the Declaration of Helsinki. Due to the retrospective nature of the study and the use of fully anonymised secondary data, the requirement for informed consent was waived. 2. Patient Selection Clinical records of patients treated with implant-supported prostheses between January 2018 and July 2022 were reviewed. Patients were included if they presented a mechanical complication requiring removal of the prosthesis and laboratory repair. Complications resolved chairside, biological complications, implant failures, and incomplete records were excluded. 3. Variables Prosthesis type was classified as: Single implant-supported crown (SISC) Implant-supported fixed partial denture (FPD) Implant-supported full-arch fixed prosthesis (FAFP) Bar-retained implant-supported overdenture (BR-IOD) Implant-supported overdenture with individual attachments (IA-IOD) Mechanical complications were categorised as material fracture, lack of adaptation, persistent crown loosening, decementation, and tooth wear. Complication severity was classified as medium or high according to the extent of laboratory intervention required. Laboratory working time was defined as the number of days between dispatch of the prosthesis to the laboratory and its return to the clinic. 4. Statistical Analysis Descriptive statistics were used to summarise prosthesis types and complication categories. Associations between categorical variables were analysed using chi-square tests. Differences in laboratory working time were analysed using non-parametric tests. Multivariable regression analyses were conducted to identify independent predictors of prolonged laboratory working time. Statistical significance was set at p &lt; 0.05.

## Results

**Figure 1:**
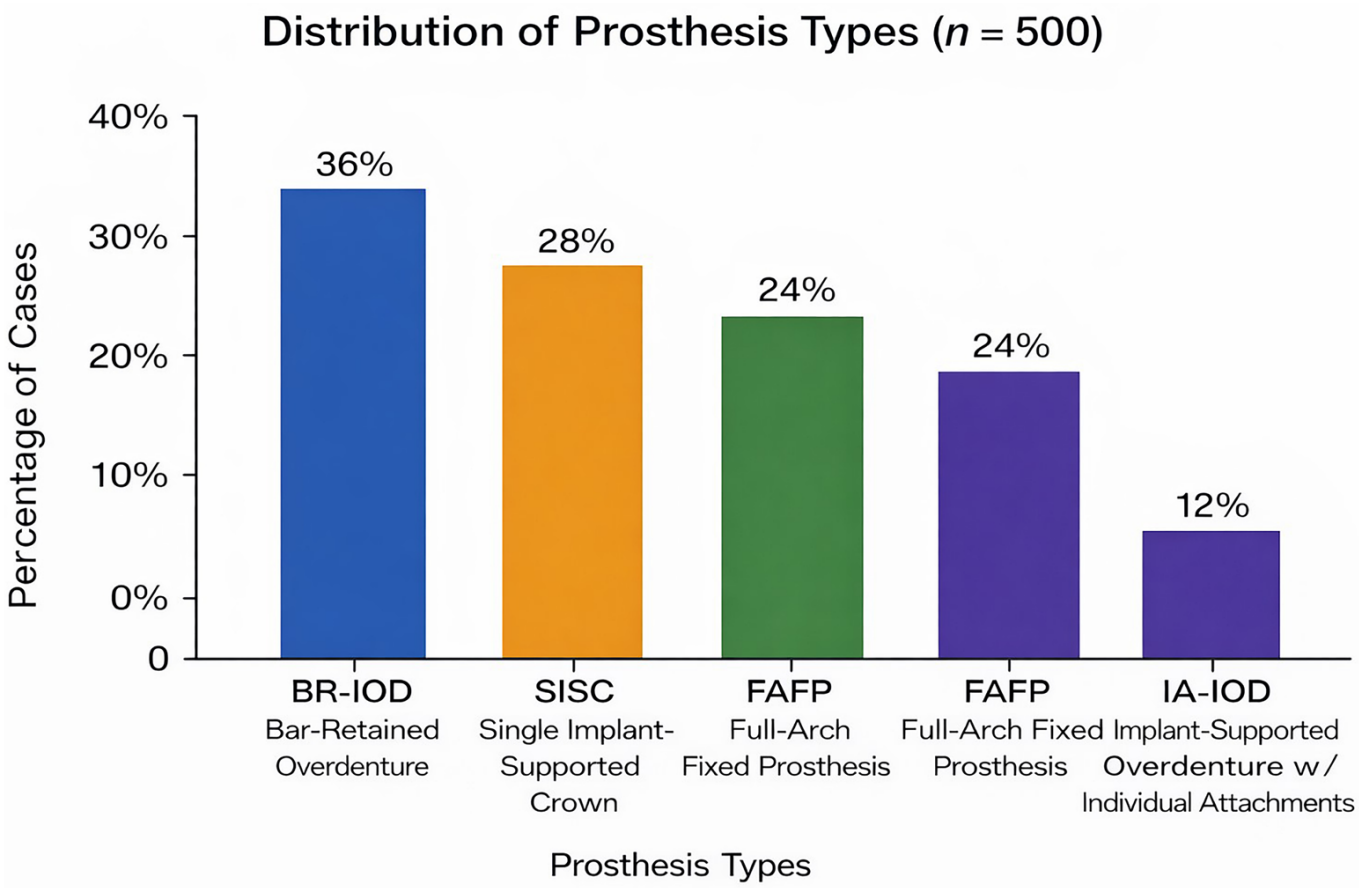
Distribution of Prosthesis Types (n=500).

1. Prosthesis Type Distribution The distribution of prosthesis types among cases requiring laboratory repair is shown in Fig. 1.

1

**Figure 2:**
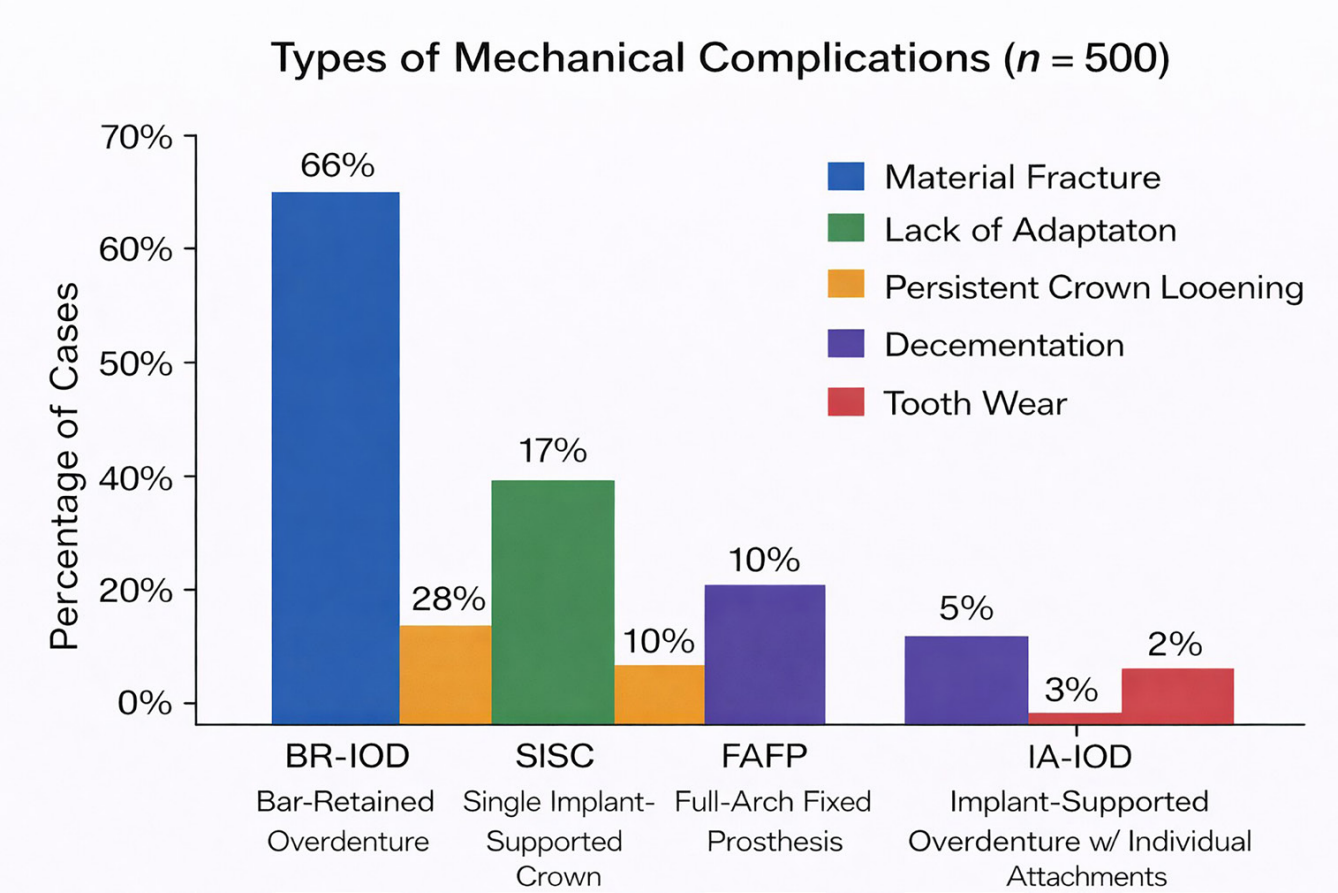
Types of Mechanical Complications (n=500).

Bar-retained implant-supported overdentures accounted for 36% of cases, followed by single implant-supported crowns (28%), implant-supported full-arch fixed prostheses (24%), and implant-supported overdentures with individual attachments (12%). 2. Mechanical Complication Categories The distribution of mechanical complication categories is presented in Fig. 2.

2

**Figure 3:**
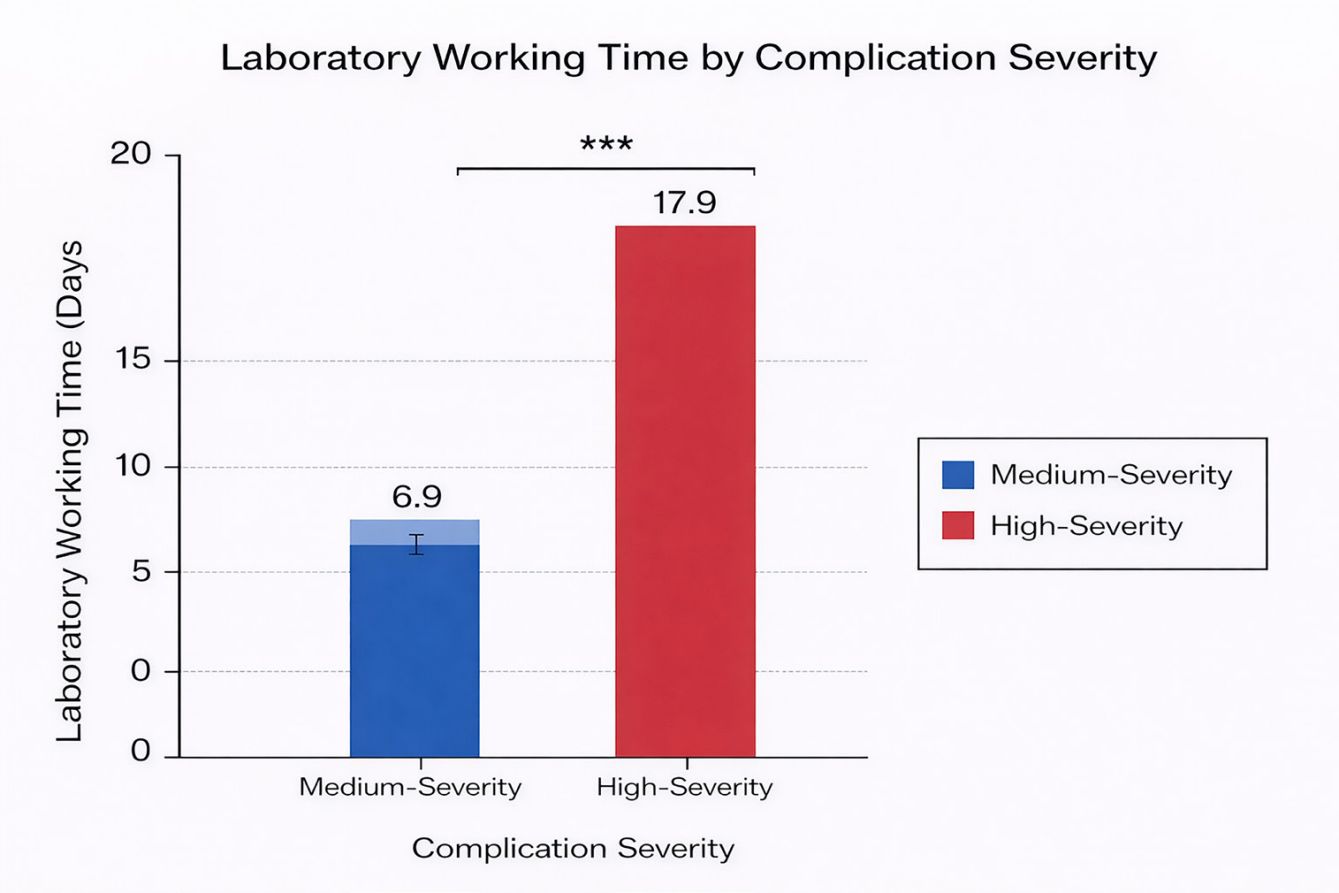
Laboratory Working Time by Complication Severety.

Material fracture was the most frequent complication (66%), followed by lack of adaptation (17%). Persistent crown loosening, decementation, and tooth wear were less frequent. 3. Association Between Prosthesis Type and Complication Category The association between prosthesis type and mechanical complication category is illustrated in Fig. 3.

3

**Figure 4:**
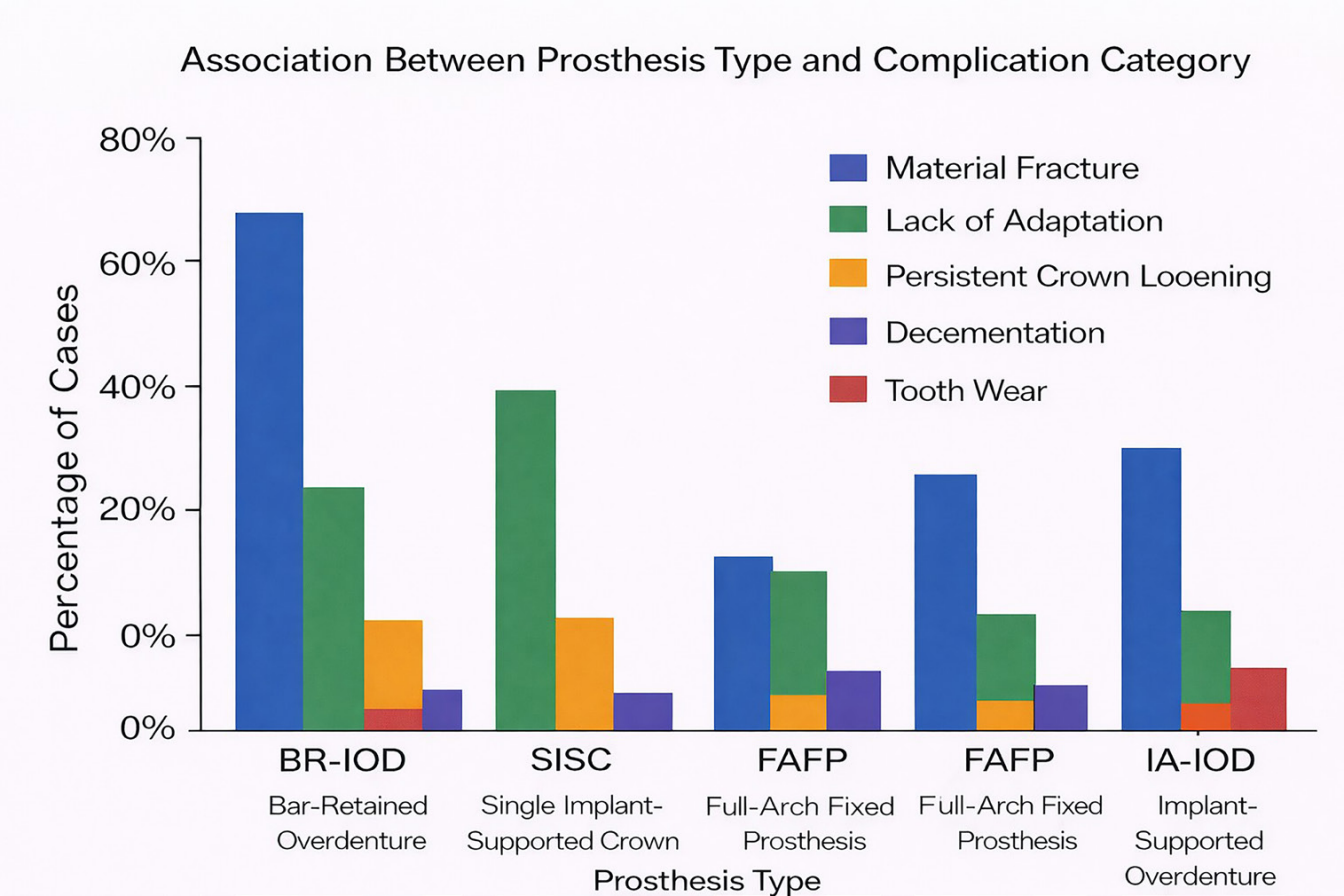
Association Between Prosthesis Type and Complication Category.

Bar-retained implant-supported overdentures were predominantly affected by material fracture, whereas single implant-supported crowns were more frequently associated with lack of adaptation. Implant-supported full-arch fixed prostheses showed a heterogeneous distribution of complications. 4. Laboratory Working Time Laboratory working time according to complication severity is shown in Fig. 4.

4

High-severity complications required significantly longer laboratory working time (mean 17.9 days) compared with medium-severity complications (mean 6.9 days; p &lt; 0.001).

## Discussion

This retrospective study analysed mechanical complications requiring dental laboratory intervention in implant-supported prostheses treated in a university-based clinical environment. By focusing exclusively on complications necessitating laboratory repair, the study addresses an operational aspect of prosthetic maintenance relevant to clinical workflow and patient management. Material fracture was the most frequent complication, particularly in bar-retained implant-supported overdentures, consistent with previous reports describing high fracture rates in removable implant-supported prostheses (1 , 5 , 8). Biomechanical factors such as flexural deformation of the acrylic base and stress concentration at the bar-resin interface may contribute to material fatigue over time (9 , 10). Single implant-supported crowns were more frequently associated with lack of adaptation. This finding is consistent with studies highlighting the technical sensitivity of single-unit implant restorations, where marginal and internal fit are critical for long-term performance (11 - 13). Manufacturing technique and cementation protocol may influence adaptation and mechanical stability (12 , 14). Laboratory working time was strongly influenced by complication severity. High-severity complications required significantly longer repair times, in agreement with studies evaluating maintenance interventions in implant prosthodontics (4 , 15). Such delays may affect patient satisfaction and require interim clinical solutions. The university-based setting may introduce variability related to operator experience, including undergraduate students, postgraduate trainees, and faculty clinicians. Operator-related variability has been reported as a potential confounder in prosthetic outcomes (7 , 16). In addition, changes in materials and laboratory workflows during the study period may have contributed to heterogeneity (6 , 14). The results should be interpreted as descriptive, characterising the distribution and operational impact of mechanical complications requiring laboratory intervention rather than providing incidence or risk estimates. Limitations The retrospective design limited control over data collection and standardisation. Variables such as antagonist dentition, prosthesis location, prosthesis age, number of previous repairs, and history of earlier complications were not consistently available and could not be included. The single-centre, university-based setting may limit external validity. Operator-related variability could not be controlled and may have influenced complication patterns and management. The analysis was restricted to cases presenting mechanical complications requiring laboratory intervention. As denominators representing the total number of prostheses placed were not available, incidence or comparative risk could not be calculated. Heterogeneity in prosthesis design and complication type limited statistical power for some analyses. Laboratory-related factors, including technician experience and manufacturing processes, were not fully controlled and may have influenced repair duration.

## Conclusions

Mechanical complications requiring laboratory intervention differed according to prosthesis design and significantly influenced laboratory working time. Bar-retained implant-supported overdentures were predominantly affected by material fracture, whereas single implant-supported crowns more frequently presented adaptation-related complications. Complication severity was strongly associated with laboratory repair duration.

## Data Availability

The data analysed in this study are available from the corresponding author upon reasonable request.
